# Distally based Chinese forearm flap in preventing impending necrosis from ring entrapment: Surgical case report

**DOI:** 10.1016/j.ijscr.2024.109253

**Published:** 2024-01-20

**Authors:** Meirizal, Muhammad Fadhil Wasi Pradipta, Mohammad Yossan Yasykur

**Affiliations:** Dept. of Orthopedic and Traumatology Sardjito General Hospital/Faculty of Medicine, Public Health and Nursing, Universitas Gadjah Mada, Yogyakarta, Indonesia

**Keywords:** Ring entrapment, Chinese forearm flap, Impending necrosis, Soft tissue coverage, Surgica case report

## Abstract

**Introduction:**

Ring entrapment is a medical condition that can lead to severe consequences, including nerve damage, ischemia, and impending necrosis. The condition often necessitates surgical intervention to prevent complications such as amputation.

**Case presentation:**

We report a case of a 49-year-old male with impending necrosis of the right little finger due to steel ring entrapment. The patient presented with severe edema, signs of infection, and undetected finger saturation on pulse oximetry.

**Discussion:**

The Distally Based Chinese Forearm Flap (CFA) was used for soft tissue coverage, which has advantages like reliable vascularization and long pedicle rotation. The CFA flap has shown to be effective in restoring perfusion to the distal tissue and facilitating early wound closure.

**Conclusion:**

The use of CFA in this case resulted in a viable flap and good finger function, demonstrating its effectiveness in managing impending necrosis due to ring entrapment.

## Highlights

• Unique management of ring entrapment in the little finger, averting amputation outcome, and successfully preventing necrosis.
• The use of a Distally Based Chinese Forearm Flap (CFA) provided effective healing and functionality.
• The chosen flap offered adequate vascularization and a thin, non-bulky skin paddle, vital for sufficient wound closure without excessive tissue volume.

## 1 Introduction

Ring entrapment is by no means a negligible case, as it may damage tissue, nerves, and vasculature of the affected finger. If not promptly addressed, these cases can escalate to severe consequences, including nerve damage, ischemia, and impending necrosis that necessitates amputation. Early and precise treatment may also facilitate early wound closure and reduce the risks of wound infection, fibrosis, and scarring [1]. Ring entrapments often involve the ring finger, which contribute to 54 % decrease of grip strength when injured. Damaged ulnar digits may demand additional measures to recover strength and functionality, which could potentially hinder their ability to perform routine functional tasks [2]. It's important that this soft tissue covering not only addresses the immediate closure requirements but also considers potential future surgical interventions such as tendon repairs, arthroplasties, and bone surgeries. Reconstruction around the elbow, wrist, and hand that cannot be adequately addressed using split thickness skin grafts. This challenge becomes even greater when crucial elements such as bones, joints, tendons, nerves, and blood vessels are exposed. In these cases, the solution typically involves resorting to flap coverage techniques [3].

The challenge in impending necrosis wounds management is not only to select the appropriate flap to close the wound, but also ensuring that the chosen flap can effectively restore perfusion to the distal tissue [4]. CFA was initially introduced in the early 1980s by Yang et al [5], which subsequently gained popularity due to its versatility and numerous studies demonstrating its benefit. The technique adapted its pseudonym as the “Chinese Flap” due to the study published in Chinese literature. These advantages include the thin skin paddle, reliable vascularization by radial artery, long pedicle rotation, along with its ability to provide a good and early mobilization [6].

We present a patient with impending necrosis of the little finger of his right hand with a severe edema due to a steel ring entrapment. Attempts to cut the ring is difficult, especially due to ischemia of the finger. The challenge in this case is removing the ring and managing damaged soft tissue (impending necrosis). Attempted to treat impending necrosis wound defects using CFA as a flap method which we estimated was able to close the wound and restore the viability of the damaged tissue. The writing of this case report complied with the SCARE criteria [7].

## 2 Case presentation

**Fig. 1:**
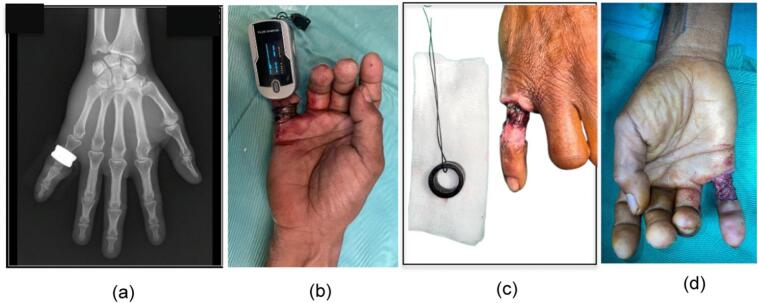
Before Chinese forearm flap (a) X–ray with ring entrapment, (b) nonviable distal tissue with undetected saturation (c) After ring removal.

We present a 49 year old male mechanic, who has no history of comorbid diseases and no routine medications came to our medical facility with a steel ring entrapped around his right little finger, a condition he had been grappling with for one week prior to his admission. Upon conducting a thorough physical examination, we observed a wound of circumferential shape on the proximal phalanx of the right little finger, which exhibited a slight cyanotic appearance indicative of impending necrosis. Accompanying this were signs of infection, pronounced edema, and superficial necrosis affecting both the extensor digitorum communis (EDC) and flexor tendons. Notably, the finger's saturation levels were undetectable when assessed using pulse oximetry (Fig. 1).

Initial endeavours to remove the entrapped ring were complicated by the ischemic condition that had developed in the finger, rendering straightforward removal unfeasible. Consequently, a surgical debridement procedure was executed to excise necrotic and non-viable tissue from the affected area. In addition to this, a debulking procedure was carried out to alleviate the severe tissue edema, thereby facilitating the subsequent removal of the entrapping steel ring. To address the need for soft tissue coverage following these interventions, we opted to employ a CFA.

**Fig. 2:**
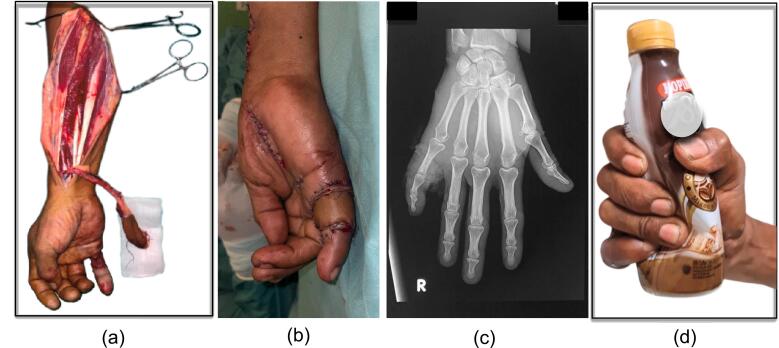
After Chinese forearm flap (a) flap harvesting (b) flap application on wound defect (c) X-ray 3 months after CFA (d) hand function 3 months after CFA.

The surgical procedure involved positioning the patient supine, mapping the radial artery, and designing the Radial Forearm Flap (RFF). A tourniquet was used during surgery for clear visibility. After debriding the recipient area, dissection was carefully carried out, prioritizing the preservation of the radial artery. The flap was then transposed to the defect site through a tunnel, ensuring no compression of the pedicle. Flap placement was optimized to prevent ischemia and venous congestion. Closure was performed with attention to tension-free suturing, and a drain was inserted as necessary (Fig. 2).

Subsequent to the surgical intervention, a comprehensive postoperative evaluation was meticulously conducted. This evaluation aimed to assess both the viability of the CFA and the functional integrity of the patient's surgically treated little finger. To this end, a range of clinical metrics were employed, serving as quantitative measures to gauge the overall success and effectiveness of the surgical intervention (Fig. 2).

Three months subsequent to the surgical procedure, the patient demonstrated remarkable improvement in the functionality of the affected little finger. The patient was not only able to use the finger for routine daily activities but also reported no hindrance in using it for occupational tasks. Importantly, the patient's Quick Disabilities of the Arm, Shoulder and Hand (Q-DASH) score was notably favorable, registering at 9.1 (Table 1). This score is indicative of minimal disability and underscores the successful outcome of the surgical intervention, affirming the patient's ability to engage in both daily activities and work without limitations.

**Table 1:** Q-DASH Score 3 months follow up.

	No difficulty	Mild difficulty	Moderate difficulty	Severe difficulty	Unable
1. Open a tight or new jar	+1	***+2***	+3	+4	+5
2. Do heavy household chores (e.g. wash, walls, floors, etc).	***+1***	+2	+3	+4	+5
3. Carry a shopping bag or briefcase.	+1	***+2***	+3	+4	+5
4. Wash your back	***+1***	+2	+3	+4	+5
5. Use a knife to cut food	***+1***	+2	+3	+4	+5
6. Recreational activities in which you take some force or impact through your arm, shoulder, or hand (e.g. Golf, hammering, tennis, etc)	***+1***	+2	+3	+4	+5


	Not at all	Slightly	Moderately	Quite a bit	Extremely
7. During the past week, to what extent has your arm, shoulder, or hand problem interfered with your normal social activities with family, friends, neighbors, or groups?	***+1***	+2	+3	+4	+5


	Not limited at all	Slightly limited	Moderately limited	Very limited	Unable
8. During the past week, were you limited in your work or other regular daily activities as a result of your arm, shoulder, or hand problem?	+1	***+2***	+3	+4	+5


	None	Mild	Moderate	Severe	Extreme
9. In the last week, please rate the severity of arm, shoulder, or hand pain	***+1***	+2	+3	+4	+5
10. In the last week, please rate the severity of tingling (pins and needles) in your arm, shoulder, or hand.	+1	***+2***	+3	+4	+5


	No difficulty	Mild difficulty	Moderate difficulty	Severe difficulty	Cannot sleep
11. During the past week, how much difficulty have you had sleeping because of the pain in your arm, shoulder, or hand?	***+1***	+2	+3	+4	+5
Q-DASH Score	***9.1/100= 9.1%***				

Data in bold and italic shows the patient's score.

## 3 Discussion

Finger injuries caused by trapped rings have been linked to various factors, including infection, skin issues, allergies, or simply ring size incompatibility. Though these cases seem trivial, delayed treatment may lead to severe complications, such as nerve damage, ischemia, and possibly even gangrene of the affected finger that leads to amputation [1]. Given the significant contribution of ulnar digits to overall grip strength, losing one or both digits will produce significant loss of overall grip strength and significant negative impact on functional activities. Methot et al. [2] reported an average 33 % decrease of grip strength after the little finger was restricted, which increased to an average of 54 % when both the little and ring fingers were restricted.

The finger should initially be examined for presence of edema and ischemia on the finger that indicate an urgent ring removal. Ischemia may manifest as a shooting pain, extended capillary refill time, cyanosis, and inability to perform a 2-point discrimination. Negative findings must be followed by secondary examination for fractures, open wounds, or advanced arthritis. This assessment will aid in determining the appropriate approach for removing the ring, which introduces another challenge in itself. Manual ring cutters may cut rings made from gold, silver, copper, or plastic, but the saw may be damaged when attempting to cut extremely hard materials such as steel, tungsten carbide, or titanium. These materials require an additional device, such as electric hand saws [8]. Aside from that, patients may present to the hospital after ill-judged attempts to remove the ring, making retrieval impossible due to distal bunching of the skin and soft tissue. It may also result in further problems of circumferential degloving and neurovascular defect [9].

Challenges arise when dealing with soft tissue repair in hand injuries requiring flap cover, primarily due to the lack of nearby tissue resources. Historically, Lister et al. [10] and McGregor [11] reported the use of distant flaps, which may restrict the mobility of the limb as it requires attachment of the injured limb to another body part. However, techniques to cover hand and forearm soft tissue defects are continuously progressing over time, especially with the development of free flaps and microsurgery. Several studies have reported a satisfactory outcome with utilization of groin flap [12], paraumbilical flap [13], Dorsal Intermetacarpal flap [14], Posterior Interosseus flap [15], Infraclavicular flap [16], cross flap [17], and Chinese Forearm (CFA) flap.

CFA flap is a type of fasciocutaneous flap that receives its vascularization through the radial artery and its accompanying venae comitantes enclosed within the lateral intermuscular septum. Although this technique was initially introduced by Yang et al. [18] to repair injuries on the opposite side, subsequent findings demonstrated that the same flap could be connected to the radial artery, venae comitantes, and cephalic vein to serve as an independent flap, covering the hand on the same side. Nowadays, CFA stands out as an exceptional and incredibly adaptable technique due to its ability to offer an ample donor region, high-quality skin texture, minimal subcutaneous tissue, and most importantly, its feasibility for a single-stage operation. The donor flap contains sensory nerves and rich vascularization links between the flap, radius, and forearm tendons. Gang [19] reported five successful cases of hand and forearm injuries with this technique. Another study by Biemer et al. [20] incorporated a bone segment as an osteocutaneous flap in thumb reconstruction, which later achieved a good functional outcome.

In managing the impending necrosis in our patient's case, CFA was selected for its superior vascularization, as emphasized by Yang et al. [18] and Biemer et al. [20]. This choice was pivotal due to the urgent need for robust blood supply to the affected area. Unlike typical applications of the CFA flap, our case required sacrificing the radial artery, a decision that balanced immediate tissue needs against long-term vascular considerations.

The radial forearm flap, known for its larger and longer caliber, was ideal for reaching the affected pinkie finger, a less common application area compared to its usual usage on the dorsal hand or thumb. This adaptability, coupled with its thin skin paddle and reliable vascularization, made it a standout choice. Furthermore, the flap's ability to provide early mobilization was a significant factor in our decision-making process. Despite the technical challenges, our successful implementation underscores the flap's versatility in emergency scenarios and contributes to its growing evidence as a critical tool in reconstructive surgery.

This case not only demonstrates the efficacy of the CFA flap in unusual anatomical locations but also highlights the importance of tailoring surgical strategies to specific patient needs. Our experience adds to the existing literature by showcasing the flap's potential in complex, urgent cases, providing valuable insights for future applications in similar scenarios.

Several drawbacks have been reported, including reduced tolerance to cold, altered sensitivity, aesthetic concerns, upper limb claudication, as well as shifts in both motor function and temperature perception. It is still widely discussed whether sacrifice of the radial artery to perform CFA flap negatively impacts the patient, albeit being supported by limited research and literature. In routine practice, some professionals even discourage the use of CFA flap due to concerns on sacrificing the radial artery and compromising hand circulation, despite the overall arterial system of the limb remaining intact [23]. In our case, despite these general concerns, our patient showed no such drawbacks after surgery. This positive outcome aligns with Costa et al.'s findings [21] that complications are often trauma-related rather than due to the arterial sacrifice. Our patient's successful recovery suggests that with careful surgical planning and patient selection, the benefits of the CFA flap can be realized while minimizing potential adverse effects. Nevertheless, ultrasound investigations by Ciria-Llorens et al [24] revealed that the overall blood flow rate to the hand remained unchanged even after the radial artery was lost. Gaudino et al. even reported that the thickness of both intima and media layers of the ulnar artery wall was greater in the harvested arm compared to the contralateral part [25]. While a minor reduction in the transcutaneous partial pressure of oxygen of the fingertips during exercise was observed, none of these patients displayed signs and symptoms of ischemia. Study by Costa et al [21] also showed complications of CFA flap have mostly been linked to the trauma process without any evidence that sacrificing the radial artery was responsible.

## 4 Conclusion

Wound defects on the hands require fast and precise treatment. Flaps are needed to close the wound, choosing the right type of flap can support patient healing. CFA has its own advantages compared to other flaps, especially in cases which need flaps with minimal bulky and long rotation which is suitable to repair distal perfusion. This case report shows satisfactory results of CFA, however, further research is needed to determine the role of CFA in treating impending necrosis wounds.

## Consent

Written informed consent was obtained from the patient for publication of this case report and any accompanying images. A copy of the written consent is available for review by the Editor-in-Chief of this journal.

## Ethical approval

This case report doesn't require ethical approval based on the Universitas Gadjah Mada research ethics committee's guidelines. It focuses on a patient's treatment and medical care, not research. Our institution's ethics committee confirmed that this report aligns with routine clinical practice and doesn't involve experimental interventions or additional data collection. We're ready to provide more information if needed, underscoring our commitment to ethical practices.

## Funding

This paper did not receive any specific grant from funding agencies in the public, commercial, or non-profit sectors.

## Author contribution

Conceptualization: M.M., M.F.W.P., M.Y.Y.

Data Curation: M.M., M.F.W.P., M.Y.Y.

Formal Analysis: M.M., M.F.W.P., M.Y.Y.

Funding acquisition: Not applicable

Investigation: M.M., M.F.W.P., M.Y.Y.

Project Administration: M.M., M.F.W.P., M.Y.Y.

Resources: M.M., M.F.W.P., M.Y.Y.

Software: Not applicable

Supervision: M.M., M.F.W.P., M.Y.Y.

Validation: M.M., M.F.W.P., M.Y.Y.

Visualization: M.M., M.F.W.P., M.Y.Y.

Writing-original draft preparation: M.M., M.F.W.P., M.Y.Y.

Writing-review and editing: M.M., M.F.W.P., M.Y.Y.

All authors have read and agreed to the published version of the manuscript.

## Guarantor

Meirizal.

## Research registration number

This study is not “First In Man” studies.

## Conflict of interest statement

The authors have no conflict of interest.

## Data Availability

Supporting data will be available upon reasonable request.
